# Supplementary material for: Neural alignment predicts learning outcomes in students taking an introduction to computer science course
Source: Nat Commun. 2021 Mar 26;12:1922. doi: 10.1038/s41467-021-22202-3 (PMC7997890; doi:10.1038/s41467-021-22202-3)
Supplement: Supplementary file 1 — Supplementary Information [file 41467_2021_22202_MOESM1_ESM.pdf]

Supplementary Information

Supplementary Results

Prediction of learning outcomes improves with number of lecture segments

We performed a power analysis across lectures to determine the amount of neural data required in order to obtain robust correlations with exam scores (i.e. how early in the course we could predict learning outcomes). This was motivated by our desire to inform future studies and applications of our measures to real-world scenarios, where resource optimization (i.e. less scanning) may be desired. To this end, we first obtained alignment-to-class values for each student in each lecture segment (21 in total). Then, we correlated exam scores with alignment in the first segment, the first two segments, and so forth until information from all segments was accumulated. This has allowed us to examine changes in score prediction quality due to the accumulation of information across lectures. An ROI analysis showed that, in the hippocampus, prediction quality increased steadily as more data was added, and afforded significant prediction after a single scan (Supplementary Fig. 1). To test whether this was the case across the cortex, we calculated a “Stable Prediction Index” for every voxel in the brain using searchlight (see Methods). On this index, a low number corresponded to regions where few data points were required to achieve significant correlation with behavior (i.e. early prediction), and a higher number to regions where more data points were required (i.e. late prediction). We found significant variance within and across cortical regions. Thus, while alignment-to-class in some parts of the angular gyrus afforded early prediction, only late prediction was possible in other parts (i.e. given the entire dataset).

Correlation between alignment measures during the exam is robust to response length

We examined the link between alignment-to-class and alignment-to-experts during the exam while controlling for response length. While we found a strong positive correlation between these alignment measures across ROIS during both recaps and the exam (Table 3), neural responses during the exam could conceivably be affected by response length as discussed in the main text. To address this, we used a within-participant regression model to predict alignment from answer length. This model yielded a residual error term for each question (“residual score”, predicted alignment minus true alignment). We then correlated residual alignment-to-class and residual alignment-to-experts during the exam (Supplementary Table 2). We found that across ROIs, correlation values were somewhat higher in the control analysis, arguing against a contribution of response length to the effects shown in Table 3.

Supplementary Figures

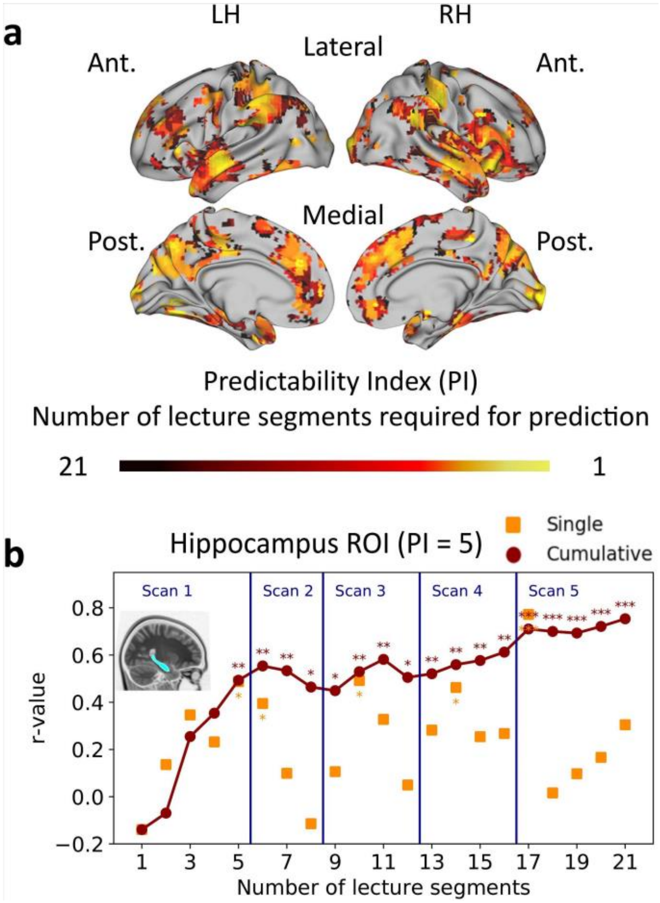

Supplementary Figure 1. Variance across the brain in the number of lectures required for performance prediction. a. Searchlight analysis results. Per-voxel "predictability index" values shown. Note the low index scores across major DMN nodes, indicating prediction of exam score can be achieved with a small number of lecture segments. **b**. Number of lectures required for stable prediction in the hippocampus. Yellow rectangles, prediction result for individual lecture segments (correlation between exam scores and alignment-to-class in that segment). Brown line, prediction of exam score from data accumulated over lecture segments. Asterisks denote significant correlation (one-sided permutation test, uncorrected), * p<0.05, ** p<0.01.

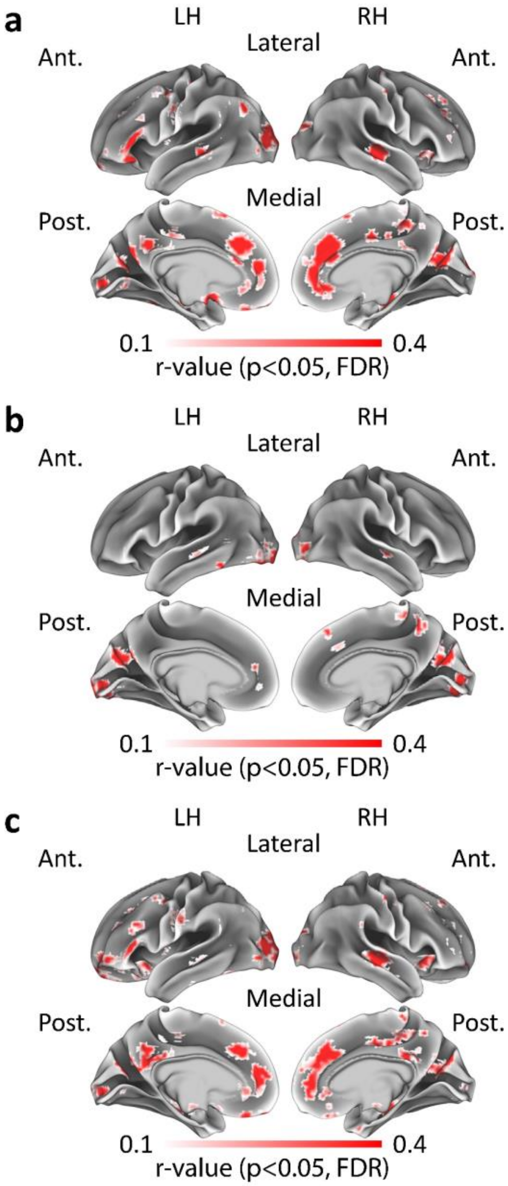

Supplementary Figure 2. "Same-question" and "Knowledge structure" effects controlled for response length. Searchlight analysis results shown. Voxels showing significant correlation are shown in color (one-sided permutation test, $p<0.05$, corrected). **a**. Correlation between same-question alignment-to-class and exam score, controlled for response length. **b**. Correlation between same-question alignment-to-experts and exam score, controlled for response length. **c**. Correlation between "knowledge structure" alignment-to-class and exam score, controlled for response length. Note the close correspondence between these maps and the results of the original analyses in Fig. 4D and Fig.5C. LH, left hemisphere, RH, right hemisphere, Ant., anterior, Post., posterior.

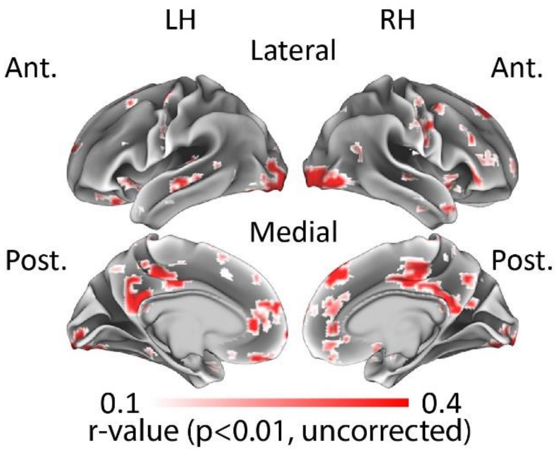

Supplementary Figure 3. Correlation of “knowledge structure” alignment-to-experts and performance.

Searchlight analysis results shown. Map thresholded using a liberal statistical threshold (one-sided permutation test, $p<0.01$, uncorrected). No voxels survived multiple comparisons correction ($p<0.05$, FDR). Note the qualitative similarities to knowledge structure alignment-to-class results in medial cortical structures (Fig. 5C). LH, left hemisphere, RH, right hemisphere, Ant., anterior, Post., posterior.

Supplementary Tables

Region of Interest (ROI)	Lectures		
	Corr. exam score and alignment-to-class (spatial) computed in 10s time bins	Corr. exam score and alignment-to-class (spatial) computed in each TR	Corr. exam score and alignment-to-class (temporal)
Angular Gyrus	0.51*	0.46*	0.59*
Ant. Cingulate (ACC)	0.50*	0.42 (n.s.)	0.36 (n.s.)
Hippocampus	0.59*	0.26 (n.s.)	0.61*
Post. Sup. Temporal Gyrus	0.30 (n.s.)	0.30 (n.s.)	0.51*
Precuneus	0.59*	0.59*	0.55*
Amygdala	0.35 (n.s.)	0.30 (n.s.)	0.49*
Early Auditory	0.54*	0.51*	0.39*
Early Visual	0.41 (n.s.)	0.42 (n.s.)	0.55*

Supplementary Table 1. Prediction of exam score from alignment-to-class during lectures using alternative alignment-to-class measures. Left and middle columns, correlation of exam score and alignment-to-class computed using 10-second time bins (left) and with no binning (middle). Right column, correlation of exam score with a temporal measure of alignment-to-class (Inter-Subject Correlation, ISC). Results are shown in DMN ROIs as well as in control regions (text shaded in gray) in sensory cortex (visual, intracalcarine cortex; auditory, Heschl's gyrus) and subcortex (amygdala). Asterisks denote significant correlation (one-sided permutation test, $p<0.05$, FDR corrected across ROIs). * $p<0.05$, ** $p<0.01$, n.s., not significant.

Region of Interest (ROI)	Corr. Alignment-to-class and alignment-to-experts
	Exam
Angular Gyrus	0.71**
Ant. Cingulate (ACC)	0.88**
Hippocampus	0.49**
Post. Sup. Temporal Gyrus	0.51**
Precuneus	0.71**
Amygdala	0.42**
Early Auditory	0.76**
Early Visual	0.56**

Supplementary Table 2. Alignment-to-experts is positively correlated with alignment-to-class in response length control. Correlation between alignment-to-class (controlled for response length) and alignment-to-experts (controlled for response length) during the exam is shown. Results are shown in DMN ROIs as well as in control regions (text shaded in gray) in sensory cortex (visual, intracalcarine cortex; auditory, Heschl's gyrus) and in subcortex (amygdala). Asterisks denote significant correlation (one-sided permutation test, $p<0.05$, FDR corrected across ROIs). * $p<0.05$, ** $p<0.01$, n.s., not significant.

Supplementary Text

Exam questions

1. What is recursion?
2. Suppose that you have written a recursive method in Java. When you run a program that calls this method, the program crashes with a StackOverflowError. What is the most likely bug in your method?
3. What is the difference between a static variable and an instance variable in Java? (Instance variables are sometimes called state variables, if that terminology is more familiar to you.)
4. What are generics in Java?
(If you aren't familiar with the term, consider the two instances of String in the declaration List<String> s = new ArrayList<String>();).
Why do we design classes to be parameterized in this way?
5. What are the restrictions on the types that can be used as a generic parameter? Why does this restriction exist?
6. What is a linked list? What differentiates the variations of singly-linked, doubly-linked, and circular linked lists?
7. What is the time complexity (for instance, in
big-O or ~ notation) for inserting an arbitrary item into a conventional linked list?
8. What are the key differences between stacks and queues?
9. What is a balanced binary search tree? (It may be easiest to answer this question one word at a time, backwards: what is a tree, what is a search tree, etc.)
10. Are there computational problems that are impossible to write an algorithm to solve? If so, describe one or more of them. If not, explain why.
11. Briefly describe the open problem "Does P = NP?".
12. What is a deterministic finite automaton (DFA)? (You might have seen this structure called finite state machine (FSM)). How is this structure related to a regular expression (RE)?
13. What is a Turing machine? What is the key difference between a Turing machine and a DFA?
14. What is 2's complement? What decimal integer value is represented in 8-bit 2's complement by 11100011 ?
15. Suppose you have a bitstring x. What integer value results when you XOR x with itself? (XOR is the operation ^ in Java.)
16. Briefly describe a NAND gate or circuit. It may be useful to describe the truth table for a binary NAND operation.
