# Supplementary material for: Neural alignment predicts learning outcomes in students taking an introduction to computer science course
Source: Nat Commun. 2021 Mar 26;12:1922. doi: 10.1038/s41467-021-22202-3 (PMC7997890; doi:10.1038/s41467-021-22202-3)
Supplement: Supplementary file 3 — Reporting Summary [file 41467_2021_22202_MOESM3_ESM.pdf]

nature research

Corresponding author(s): Meir Meshulam

Last updated by author(s): Dec 25, 2020

Reporting Summary

Nature Research wishes to improve the reproducibility of the work that we publish. This form provides structure for consistency and transparency in reporting. For further information on Nature Research policies, see our Editorial Policies and the Editorial Policy Checklist.

Statistics

For all statistical analyses, confirm that the following items are present in the figure legend, table legend, main text, or Methods section.

n/a	Confirmed	
☐	☒	The exact sample size (*n*) for each experimental group/condition, given as a discrete number and unit of measurement
☐	☒	A statement on whether measurements were taken from distinct samples or whether the same sample was measured repeatedly
☐	☒	The statistical test(s) used AND whether they are one- or two-sided<br>*Only common tests should be described solely by name; describe more complex techniques in the Methods section.*
☐	☒	A description of all covariates tested
☐	☒	A description of any assumptions or corrections, such as tests of normality and adjustment for multiple comparisons
☐	☒	A full description of the statistical parameters including central tendency (e.g. means) or other basic estimates (e.g. regression coefficient) AND variation (e.g. standard deviation) or associated estimates of uncertainty (e.g. confidence intervals)
☐	☒	For null hypothesis testing, the test statistic (e.g. F, t, r) with confidence intervals, effect sizes, degrees of freedom and P value noted<br>*Give P values as exact values whenever suitable.*
☒	☐	For Bayesian analysis, information on the choice of priors and Markov chain Monte Carlo settings
☒	☐	For hierarchical and complex designs, identification of the appropriate level for tests and full reporting of outcomes
☐	☒	Estimates of effect sizes (e.g. Cohen's *d*, Pearson's *r*), indicating how they were calculated

Our web collection on statistics for biologists contains articles on many of the points above.

Software and code

Policy information about availability of computer code

Data collection	PsychoPy2 (https://www.psychopy.org/)
Data analysis	FSL 6.0.1 (http://fsl.fmrib.ox.ac.uk/fsl), Brain Imaging Analysis Kit (http://brainiak.org), and custom code in Python 3 (www.python.org) and R 3.5.1 (www.r-project.org). Analysis code is available on GitHub (https://github.com/me-sh/think_like_an_expert_paper).

For manuscripts utilizing custom algorithms or software that are central to the research but not yet described in published literature, software must be made available to editors and reviewers. We strongly encourage code deposition in a community repository (e.g. GitHub). See the Nature Research guidelines for submitting code & software for further information.

Data

Policy information about availability of data

All manuscripts must include a data availability statement. This statement should provide the following information, where applicable:

- Accession codes, unique identifiers, or web links for publicly available datasets
- A list of figures that have associated raw data
- A description of any restrictions on data availability

The imaging data that support the findings of this study are available in OpenNeuro with the identifier “doi: 10.18112/openneuro.ds003233.v1.2.0” (https://openneuro.org/datasets/ds003233/versions/1.2.0).

Field-specific reporting

Please select the one below that is the best fit for your research. If you are not sure, read the appropriate sections before making your selection.

☐ Life sciences ☒ Behavioural & social sciences ☐ Ecological, evolutionary & environmental sciences

For a reference copy of the document with all sections, see nature.com/documents/nr-reporting-summary-flat.pdf

Behavioural & social sciences study design

All studies must disclose on these points even when the disclosure is negative.

Study description	In this work, we introduce a neural approach to predicting and assessing learning outcomes in a real-life setting. We show that learning is mirrored in neural alignment: the degree to which an individual learner's neural representations match those of experts, as well as those of other learners.
Research sample	Twenty-four "student" and five "expert" participants were recruited for the study (11 female, mean age 20.4 years, s.d. 3.8). Students were enrolled in COS 126: Computer Science - An Interdisciplinary Approach. Experts all had an undergraduate or graduate degree in computer science. The sampling rationale for student participants was to obtain a representative sample of the population of course students.
Sampling strategy	Random sampling was used. No statistical methods were used to predetermine sample sizes. Our student sample size is similar to those reported in previous publications from our group (Chen, J. et al. Shared memories reveal shared structure in neural activity across individuals. Nat Neurosci 20, 115–125 (2017); Honey, C. J., Thompson, C. R., Lerner, Y. & Hasson, U. Not lost in translation: neural responses shared across languages. J Neurosci 32, 15277–83 (2012); Regev, M., Honey, C. J., Simony, E. & Hasson, U. Selective and invariant neural responses to spoken and written narratives. J Neurosci 33, 15978–88 (2013).
Data collection	Imaging data was collected using 3T functional MRI. Responses to open exam questions were collected using pen and paper (pre-exam, outside the scanner) and audio recordings (post-exam, inside the scanner). All data was collected in testing rooms in the presence of the research team only (M.M., L.H., H.H., Y-F.L. and/or M.N). The research team was not blind to the study hypotheses during data collection.
Timing	February-June, 2018
Data exclusions	Four students dropped the course and were excluded from the experiment according a list of pre-established criteria.
Non-participation	No participants declined participation.
Randomization	Student (undergrads) and expert (grads and postdoc) participants were allocated to groups according to their level of education.

Reporting for specific materials, systems and methods

We require information from authors about some types of materials, experimental systems and methods used in many studies. Here, indicate whether each material, system or method listed is relevant to your study. If you are not sure if a list item applies to your research, read the appropriate section before selecting a response.

Materials & experimental systems

n/a	Involved in the study	
☒	☐	Antibodies
☒	☐	Eukaryotic cell lines
☒	☐	Palaeontology and archaeology
☒	☐	Animals and other organisms
☐	☒	Human research participants
☒	☐	Clinical data
☒	☐	Dual use research of concern

Methods

n/a	Involved in the study	
☒	☐	ChIP-seq
☒	☐	Flow cytometry
☐	☒	MRI-based neuroimaging

Human research participants

Policy information about studies involving human research participants

Population characteristics	See above
Recruitment	We recruited 'student' participants from the Spring 2018 class of the Princeton University Computer Science Dept. Introduction to Computer Science course, and 'experts' from the CS/Engineering graduate student and postdoc pool. Participants were recruited by sending introduction emails to the course and grad students mailing lists. Participation was

fully voluntary with participants self-selecting into the study. Participation was limited to participants who were right-handed, had normal or corrected-to-normal vision and hearing, and reported no learning disabilities.

Ethics oversight

The study complied with all relevant ethical regulations for work with human participants and informed consent was obtained in accordance with experimental procedures approved by the Institutional Review Board at Princeton University, protocol #10382. No participants were of age below 18 years.

Note that full information on the approval of the study protocol must also be provided in the manuscript.

Magnetic resonance imaging

Experimental design

Design type

We used a task design. Participants watched audiovisual stimuli (video lectures) and provided verbal responses to self-paced, visually-presented exam questions.

Design specifications

Student participants underwent functional MRI scans five times during a 13-week semester while watching a subset of that week's video lectures in the scanner. Subjects were asked not to view these lecture videos online before the scans. The subset of lectures shown in each scan was approximately 40 minutes long and comprised 3-5 segments (21 segments, 197 minutes in total). On the final week of the semester, students were shown - in the scanner - five 3-minute lecture recap videos with the highlights from previous weeks, followed by a final exam (Fig. 1a). To establish a baseline, the same exam was also given to students at the beginning of the semester, in written form. Graduate "experts" underwent the final scan only, watching the recap videos from all lectures and completing the final exam.

Behavioral performance measures

Open-ended verbal exam responses in the in-scanner post-test were collected using audio recordings. Video monitoring was used to monitor participants' alertness in about 40% of scans at random (Eyelink, SR Research).

Acquisition

Imaging type(s)

Functional and structural

Field strength

3-T

Sequence & imaging parameters

MRI data were collected on two full-body scanners (Siemens Skyra and Prisma) with 64 channel head coils. Scanner-participant pairing was kept constant throughout the experiment. Functional images were acquired using a T2*-weighted echo-planar imaging (EPI) pulse sequence (TR 2000 ms, TE 28 ms, flip angle 80 deg, FOV 192 × 192 mm2, whole-brain coverage with 38 transverse slices, 3 mm3 voxels, no gap, GRAPPA iPAT 2). Anatomical images were acquired using a T1-weighted MPRAGE pulse sequence (1 mm3 resolution).

Area of acquisition

Whole-brain

Diffusion MRI ☐ Used ☒ Not used

Preprocessing

Preprocessing software

fMRI data processing was carried out using FEAT (FMRI Expert Analysis Tool) Version 6.00, part of FSL (www.fmrib.ox.ac.uk/fsl). Registration to high resolution structural images was carried out using FLIRT. Preprocessing included motion correction using MCFLIRT; slice-timing correction using Fourier-space time-series phase-shifting; non-brain removal using BET; spatial smoothing using a Gaussian kernel of FWHM 6.0mm; grand-mean intensity normalization of the entire 4D dataset by a single multiplicative factor; high-pass temporal filtering (Gaussian-weighted least-squares straight line fitting, with sigma=50.0s).

Normalization

Functional volumes were coregistred and affine transformed to a template brain.

Normalization template

MNI 152, Montreal Neurological Institute.

Noise and artifact removal

MCFLIRT motion parameters (3 translations and 3 rotations) were regressed out from functional data using linear regression.

Volume censoring

Volume censoring was not performed.

Statistical modeling & inference

Model type and settings

Pearson correlation was used to correlate variables of interest. Statistical significance was evaluated using a one-sided permutation test.

Effect(s) tested

We tested for the following positive linear correlations in the imaging data:
1) Correlation between exam scores and alignment-to-class during lectures (between-participants) (Fig.2)
2) Correlation between alignment-to-experts and alignment-to-class during lectures (between-participants) (Fig.3)
3) Correlation between alignment-to-experts and alignment-to-class during exam (between-participants) (Fig.3)
4) Correlation between exam scores and same-question alignment-to-class during exam (within-participants) (Fig.4)
5) Correlation between exam scores and same-question alignment-to-experts during exam (within-participants) (Fig.4)
6) Correlation between exam scores and "knowledge-structure" alignment-to-class during exam (within-participants) (Fig.5)
7) Correlation between exam scores and "knowledge-structure" alignment-to-experts during exam (within-participants)

(Fig.5)

Specify type of analysis: ☐ Whole brain ☐ ROI-based ☒ Both

Anatomical location(s): ROIs were determined using the probabilistic Harvard-Oxford cortical and subcortical structural atlases included with FSL. Bilateral ROIs were created by taking the union of voxels in both hemispheres. A liberal threshold of >20% probability was used.

Statistic type for inference (See Eklund et al. 2016): Voxel-wise

Correction: We controlled the false discovery rate (FDR) to correct for multiple comparisons at q = .05

Models & analysis

n/a	Involved in the study
☒	☐ Functional and/or effective connectivity
☒	☐ Graph analysis
☒	☐ Multivariate modeling or predictive analysis
